# A Semantic-Enhancement-Based Social Network User-Alignment Algorithm

**DOI:** 10.3390/e25010172

**Published:** 2023-01-15

**Authors:** Yuanhao Huang, Pengcheng Zhao, Qi Zhang, Ling Xing, Honghai Wu, Huahong Ma

**Affiliations:** 1 The College of Information Engineering, Henan University of Science and Technology, Luoyang 471023, China; 2 The School of Information Engineering, Southwest University of Science and Technology, Mianyang 621010, China

**Keywords:** social networks, user alignment, semantic enhancement, graph contrastive learning

## Abstract

User alignment can associate multiple social network accounts of the same user. It has important research implications. However, the same user has various behaviors and friends across different social networks. This will affect the accuracy of user alignment. In this paper, we aim to improve the accuracy of user alignment by reducing the semantic gap between the same user in different social networks. Therefore, we propose a semantically enhanced social network user alignment algorithm (SENUA). The algorithm performs user alignment based on user attributes, user-generated contents (UGCs), and user check-ins. The interference of local semantic noise can be reduced by mining the user’s semantic features for these three factors. In addition, we improve the algorithm’s adaptability to noise by multi-view graph-data augmentation. Too much similarity of non-aligned users can have a large negative impact on the user-alignment effect. Therefore, we optimize the embedding vectors based on multi-headed graph attention networks and multi-view contrastive learning. This can enhance the similar semantic features of the aligned users. Experimental results show that SENUA has an average improvement of 6.27% over the baseline method at hit-precision30. This shows that semantic enhancement can effectively improve user alignment.

## 1. Introduction

As different social networks offer their users distinctive functions, people tend to register accounts on several different social networks. In recent years, the number of online users on each social network has grown significantly. A huge amount of user data is generated due to users sharing and communicating on various social networks. Based on these data, researchers are able to analyze users’ behavior and the evolution of social networks, which can in turn facilitate research in areas such as community discovery [1], recommender systems [2], link prediction [3], and other related fields. However, this development of multiple social networks also brings some problems. First, cross-domain user recommendation is inaccurate because users’ behavior across different social networks is not always consistent. It is also difficult to find abnormal users and trace the abnormal sources, because malicious users tend to spread false remarks on multiple social networks. After user alignment associates a user’s multiple accounts across different social networks, comprehensive analysis of users’ behaviors on these networks can be used to solve problems such as cross-domain recommendation [4] and abnormal user detection [5]. User alignment is a basic and meaningful research, and the accuracy of alignment needs to be improved.

User alignment is also known as anchor link prediction, user identification, and social network alignment [6,7,8]. Its purpose is to associate the accounts registered by real users across different social networks. However, the differences in the same user’s features and friends across the different social networks will reduce the accuracy of user alignment, which is referred to as the semantic gap problem. Improving the effect of user alignment by reducing the semantic gap can be broken down into three aspects: (1) Accurate and comprehensive representation of user characteristics. Due to the heterogeneity between different social networks, computing user similarity based on user features and network topologies is commonly influenced by noise [9,10]. Existing methods of this kind are too biased to determine whether two users of two different social networks are the same real user by simply analyzing the users’ attributes, such as age and gender. Users’ writing patterns, personal emotion, and other semantic features can be mined through an analysis of usernames and text posts [11]. Integrated consideration of the username, user-generated content, geographic location, network topology, and other data can help mine users’ semantic features, comprehensively characterize users, and reduce the negative impacts of local feature differences on user-alignment effects [12,13,14,15]. Notably, however, user feature mining methods discussed above do not consider the reliability of data, computing overhead, and missing data problems. (2) Improving noise adaptation ability. Since the user features and network topology of the same user differ slightly from one social network to another, the noise contained in the semantic features of the user will reduce the user similarity. Feng et al. [16] achieved user alignment based on the user’s position and reduced the interference of position noise with user alignment by constructing a position encoder and trajectory encoder to calculate the user similarity. Xiao et al. [9] enhanced the noise adaptation ability of the model by adding perturbations to the data and designing a noise-adapted loss function. Xue et al. [17] proposed three noise-processing strategies: dropping, retaining, and conditional retention. Notably, the above noise-processing measures do not consider the effect of data propagation between users on noise. (3) Optimizing user-alignment effects. After user features are pre-processed, user alignment is often achieved using network representation learning. This method compares the similarity of user embedding vectors to determine whether they are the same real user, after embedding users of two social networks into the same vector space. To improve the accuracy of user alignment, many embedding optimization methods have been proposed [18,19,20]. Zhang et al. [21] and Chen et al. [22] improved the alignment effect by using a generative adversarial network to optimize the embedding representation of users. Notably, while these embedding optimization methods can improve user alignment, they do not sufficiently consider the impact of highly similar users on user alignment in the same social network and in the social network to be aligned.

To solve the above problems, we propose a semantic enhancement algorithm for social network user alignment, which enhances the semantic features of users from three aspects: semantic representation, noise adaptation, and embedding optimization. It can improve the accuracy of user alignment. (1) There are different characteristics of user attributes, UGCs, and user check-ins. First, user attributes have a low computational overhead and reflect users’ behaviors. There are more semantic features included in UGCs, such as users’ preferences and writing habits, but the data volume of pictures and videos is too large. User check-ins contain highly reliable data related to the time and place of posting. Therefore, we represent the semantic features of users at multiple levels based on user attributes, text in UGCs, and user check-ins. (2) The embedded view constructed based on semantic representations contains both feature noise and topological noise. Considering the impact of data propagation among users on user alignment, we compute the semantic centrality of users based on their influence and preferences. During graph-data augmentation, the weights of features and topologies are adaptively adjusted based on semantic centrality to highlight the important features and topologies. (3) To improve user alignment, it is necessary to optimize the embedding vector of users. Friends in the same social network have similar semantics, as do aligned users in the social network to be aligned. We aggregated the important semantic features of similar neighbors by using a multi-headed graph attention network, then used contrastive learning on the same social network views and the alignment views. This approach can reduce the semantic similarity between users in the social network view while enhancing semantic similarity between aligned users in the aligned view. The social network user-alignment effect can be effectively improved by enhancing the semantic features of users using these three aspects. The contributions of the work are summarized as follows.

- Multi-level data analysis can improve the mining of users’ semantic features. We extract meta-semantic features, specifically, users’ preferences and cities of residence from UGCs and check-ins, and then extract high-level semantic features of users from user attributes, UGCs, and check-ins, based on BERT, word2vec, and meta-graph, respectively. The semantic features of users are represented on multiple levels, which reduces the interference of local semantic noise and improves the accuracy of computing user similarity.
- The heterogeneity of different social networks introduces feature and topology noise interference into the calculation of user alignment. Since users’ influence and preferences have important impacts on semantic propagation among users, we compute the semantic centrality of users based on these two features and assign appropriate weights to the features and topologies. The model’s adaptability to noise is improved by graph-data augmentation to enhance the user-alignment effect.
- As the feature embedding vectors of the same user are not exactly the same across different social networks, the user’s embedding vector is optimized by means of semantic fusion and contrastive learning. The features of the surrounding similar neighbors are aggregated using a multi-head graph attention network to enhance the semantic features of the users themselves. Contrastive learning improves the embedding distance of users in the same social network while reducing the embedding distance of aligned users in the social network to be aligned, which ensures the accuracy of the obtained user alignment.

The remainder of this article is organized as follows. The related works are reviewed in Section 2. Subsequently, Section 3 introduces the relevant definitions and user alignment issues. The details of the SENUA algorithm are described in Section 4, followed by Section 5, which presents the experiments. Finally, Section 6 concludes this article.

## 2. Related Work

### 2.1. User Alignment

User alignment has been extensively studied. Existing approaches can be classified into three categories: user feature-based, network-topology-based, and hybrid approaches.

In user feature-based approaches, the semantic features of users are mined based on data such as user attributes and UGCs to determine whether they represent the same real user by computing the user similarity [11,16,23,24,25,26,27,28,29]. During the account registration process, the username is a required item, which enables the naming habits of users to be mined; thus, the user similarity is most widely computed based on the username. Li et al. [25] analyzed the phonetic and font similarities of Chinese usernames to achieve user alignment. To deeply mine user features, Xing et al. [30] not only analyzed the length, character features, and alphabetic features of usernames, but also mined user preferences from their posted contents to improve user-alignment accuracy.

Network-topology-based approaches compare the friend network similarity of users in the source and target networks to achieve user alignment [18,31,32,33,34,35,36,37,38]. At present, network representation learning methods are commonly used to mine network topology features [35]. This kind of method can achieve user alignment by minimizing the embedding distance after embedding the user’s network topology features into a low-latitude vector space [36,37]. However, the embedding vectors of different network topologies are not stable enough. Therefore, network topology is often combined with information propagation [39], genetic algorithms [40], community discovery [38], and generative adversarial networks [18] to enhance user-feature representations.

The user-feature-based approaches focus on the users’ personal information and the content they post. The network-topology-based approach focuses on the user’s friendships. There is complementarity or redundancy between these two different types of data. Notably, while a single method with a single type of data cannot deeply mine the semantics of users, hybrid methods that combine user features and network topologies can more fully mine the semantic features of users and thereby improve user alignment [9,12,13,14,22,41,42,43,44]. Graph neural networks are commonly used at present to fuse user features and network topologies simultaneously. These methods aggregate the feature vectors of the user’s neighbors to enhance the semantic features of the user, and subsequently determine whether two users match based on the similarity of the embedded vectors [42,44]. However, mining the semantic features of users based on graph neural networks also captures feature noise and topological noise in social networks. AFF-LP [45] uses an attention mechanism to extract network topology and temporal features in order to reduce noise interference and improve the accuracy of the algorithm. Notably, this method only considers the effect of network noise, while failing to consider the feature noise due to user feature differences. GATAL [9] removes edges to simulate network noise and randomly changes node features to simulate feature noise. After noise processing, the graph attention network is used to fuse the neighborhood features so that the algorithm can maintain good performance, even under noisy conditions. In addition, the user-alignment algorithm combines graph neural networks with generative adversarial networks to solve the problem of accuracy reduction due to semantic variability [22]. While these studies have made some progress, the noise augmentation method in users’ semantic features is random; thus, it is not adaptive to the data propagation characteristics in social networks. Accordingly, the effect on user semantic enhancement needs to be improved.

### 2.2. Text Feature Extraction

There are huge amounts of text, images, video, and other multi-source data in social networks. Image and video have a high computational overhead and difficult semantic extraction. Scholars often mine text features through natural language processing [46,47]. The text contains more semantic features, which are usually mined by two steps: sequence annotation [48] and vector embedding. Since the number and completeness of words in short and long texts differ greatly, it is more effective to annotate them at different levels [49]. Shao et al. [50] analyzed the data structure based on latent variables in random fields and constructed two frameworks for sequence annotation at the word and sentence levels, respectively. The commonly used text feature embedding methods include word2vec [51], FastText [52], BERT [53], etc. BERT is a transformer-based language representation model. It performs self-supervised training by masking parts of words to mine text features. Currently, text-embedding methods are often combined with attention mechanisms to enhance the completeness and accuracy of extracted features. Our proposed user alignment approach deeply incorporates attention mechanisms to enhance the semantic features of similar users.

### 2.3. Graph Representation Learning

Graph representation learning includes node embedding, graph neural networks, and generative graph models [54]. The node embedding contains an encoder–decoder, random wandering, and matrix decomposition. This type of method is a shallow embedding model, with which is difficult to capture the deep features of nodes. It also has limitations such as high overhead and inadequate feature mining. Graph neural networks embed user features into vector space by propagating, aggregating, and updating features between nodes. This class of methods is an end-to-end deep embedding model that can perform feature mining directly based on graph data and helps to mine deep features of nodes. Deep generative models include variational autoencoders, generative adversarial networks, and autoregressive models. Normally, this class of methods usually optimizes node vectors by confronting encoders and decoders with each other. The degree of similarity between friends has a significant impact on the accuracy of user alignment. Graph neural networks can adaptively aggregate neighboring features and enhance the user’s features. Using graph neural networks has greater benefits for user alignment.

### 2.4. Graph Contrastive Learning

Contrastive learning has already received widespread research attention and made significant achievements in many tasks, such as natural language processing [55] and computer vision [56]. In recent years, contrastive learning has been applied to graph representation learning, which is referred to as graph contrastive learning. In graph contrastive learning, multiple views are generated via graph-data augmentation, and then these nodes are embedded into the vector space by encoding and projection; finally, the embedding effect is optimized by contrastive learning. You et al. [57] designed four graph-data augmentation methods: node dropout, edge perturbation, feature masking, and subgraph sampling. Hassani et al. [58] used a diffusion kernel for data augmentation, enabling each node to sense more global information. Notably, existing graph-data augmentation methods use a uniform transformation for topologies and features, which can lead to poor performance. Therefore, Zhu et al. [59] proposed an adaptive data augmentation scheme that preserves important features and topologies during augmentation.

User alignment based on either user characteristics or network topology alone is necessarily limited. The fusion of these two types of data can effectively enhance user semantic features and improve the user-alignment effect. Considering the reliability of the data and the overhead of the algorithm, we deeply mine the semantic features of users from user attributes, UGCs, and check-ins. In addition, we propose a modified graph contrastive learning approach to achieve social network user alignment; this approach uses semantic centrality in graph-data augmentation to improve the algorithm’s self-adaptation to noise, and enhances the semantic feature similarity of aligned users via contrastive learning in multiple views.

## 3. Preliminaries

In this section, we introduce the related definitions and the user-alignment issue. The symbols used in this article and the corresponding meanings are summarized in Table 1.

### 3.1. Semantic Social Network View

Social networks contain huge amounts of user data. Based on the reliability, discernment, and data scale of these data, we selected user attributes $A_{p}$, user-generated contents $A_{c}$, and user check-in $A_{\ell }$ as the basis for discerning aligned users, which ensures that sufficient semantic information is available for the represented users. The user attributes $A_{p}$ contain the username $A_{p}^{name}$, city of residence $A_{p}^{area}$, and the user preferences $A_{p}^{pref}$. We use only the text of posts as UGCs to avoid the huge overhead associated with the task of analyzing images and videos. User check-ins refer to time and place at which a user makes a post. In this paper, we consider a semantic social network view $G=\left(U,E,A\right)$. $U=\left\{u_{1},u_{1},\cdots ,u_{n}\right\}$ represents the set of *n* nodes, and each node represents a user; $E=\left\{e_{ij}=\left(u_{i},u_{j}\right)|u_{i},u_{j}\right\}$ denotes the set of edges. This is an n∗n matrix that represents the friend relationships between *n* users. If $e_{ij}=1$, users $u_{i}$ and $u_{j}$ are friends; otherwise, they are not friends. Based on the number of edges connected to node $u_{j}$, we can get the degree of node $u_{j}$ as $\sum_{i=1}^{n}e_{ij}$. The user features *A* are represented by a triplet $A=\left\{A_{p},A_{c},A_{\ell }\right\}$; these elements, respectively, represent user attributes, user-generated contents, and user check-ins.

### 3.2. Semantic Enhancement User Alignment

**Figure 1:**
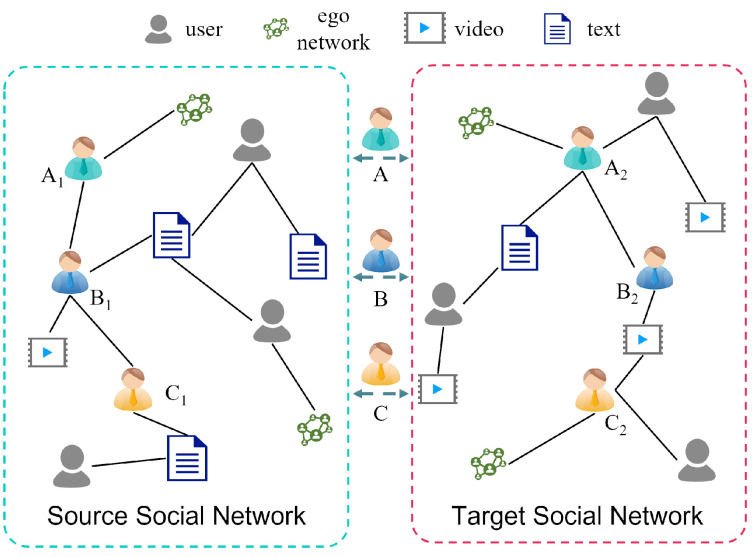
User-alignment diagram.

Typically, a user has multiple social network accounts. In this paper, we aim to solve the problem of matching social network accounts belonging to the same person, as shown in Figure 1. In order to distinguish the semantic views corresponding to different social networks, the source and target social networks to be aligned are represented by $G^{S}=\left(U^{S},E^{S},A^{S}\right)$ and $G^{T}=\left(U^{T},E^{T},A^{T}\right)$, respectively. The two views with semantic gaps include noise; we use graph-data augmentation to reduce the impact of the noise. Moreover, to improve the alignment accuracy, GAT and contrastive learning are used to enhance the semantic features of the users. Finally, we determine whether two users represent the same real user based on user similarity, that is, aligned user pairs $M=\{\left(u_{i},u_{j}\right)|u_{i}\in U^{S},u_{j}\in U^{T}\}$.

### 3.3. Multi-View Graph Contrastive Learning

Graph contrastive learning typically involves four steps: data augmentation, encoding, projection, and contrastive learning. (1) Two differing views are generated from the original view by data augmentation; (2) each view is encoded by a graph neural network; (3) the nodes of two views are mapped to the same vector space; (4) the consistency of the same node in different views is maximized by means of contrastive learning. To achieve user alignment, we propose a modified multi-view graph contrastive learning approach. Its input includes the source social network $G^{S}$ and the target social network $G^{T}$. After data augmentation is performed for both views, the view to be aligned and the augmented view are encoded as vectors. In the comparative learning stage, we not only contrast the augmented views of $G^{S}$ and $G^{T}$, respectively, but also contrast the aligned views $G^{S}$ and $G^{T}$.

In addition, to improve the effect of graph contrastive learning on user alignment in social networks, we propose a semantic centrality attention that considers the impacts of user influence and user preferences on user-alignment effects in social networks. During data augmentation and encoding, the weights are adaptively adjusted to highlight the important semantic features of users.

## 4. SENUA Algorithm

In this section, we first provide an the overview of SENUA, and then present the details of each component.

### 4.1. Overview of SENUA

**Figure 2:**
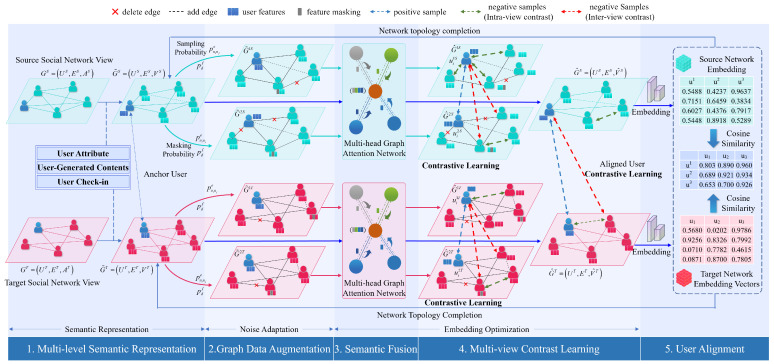
The framework of the proposed algorithm.

In this paper, we propose the SENUA algorithm to improve the accuracy of social network user alignment by enhancing the semantic features of users. The overall framework is illustrated in Figure 2, and the specific algorithm of SENUA is presented in Algorithm 1. SENUA takes as input the source social network view $G^{S}$ and the target social network view $G^{T}$ to be aligned. To improve the alignment effect, we enhance the semantic features of users in three aspects: semantic representation, noise adaptation, and embedding optimization. The process of SENUA consists of five steps. (1) Adequate user semantic feature representation can reduce the interference of the local semantic gap on global semantics. Taking user behavior, spatio-temporal information, and user relationships into account, multi-dimensional semantic features of users are extracted from user attributes, UGCs, and check-ins via semantic analysis. (2) Due to the variability between different social networks, the extracted semantic features often contain noise, which can affect the user-alignment effect. The algorithm’s noise-adaptation capability can be improved through the use of graph-data augmentation for features and topologies in multiple views. Notably, the effect of graph-data augmentation is not stable for different networks or downstream tasks. Accordingly, to improve the effectiveness of data augmentation in social network user alignment, we propose semantic centrality attention to adaptively adjust the data augmentation weights. Since the probability of data spreading among users with high influence and the same preference is higher, these users usually have more common semantic features. During graph-data augmentation, computing semantic centrality based on influence and user preferences can help to ensure that important user semantic features are retained. (3) When attempting to determine whether a user is an aligned user based on their semantic features, the key lies in how to deeply mine the similar semantic features of aligned users. Users who communicate more frequently on social networks tend to have more similar semantic features. Graph neural network-based fusion of semantic features of neighbors can thereby enhance the representations of individual users. (4) Highly similar users in the same social network can interfere with user alignment. Through the use of contrastive learning in multiple views, we not only reduce the semantic similarity between users in the same social network, but also enhance the semantic similarity between aligned users, which can optimize the feature embedding vectors of users. (5) Based on the optimized multi-view embedding vectors, user similarity is computed using the cosine distance. If the similarity reaches a threshold value, the two users are considered as aligned users. Since many operations are the same for the source social network view $G^{S}$ and the target social network view $G^{T}$, if S and T are not used to distinguish between the views in what follows, this will mean that both networks have to perform this operation.

In brief, the differences of the proposed algorithm are: (1) Multiple embedding methods are combined to fully represent user semantic features through low-level and high-level semantic feature extraction. It can reduce the influence of local noise. (2) Calculating the semantic centrality of users based on their preferences and user influence, and using it to compute the probability of topology and feature augmentation in graph-data augmentation. (3) Computing feature aggregation weights in graph attention networks based on the semantic centrality of users. (4) The application scenario of contrastive learning is extended from a single social network to multiple social networks. Enhance similarity between aligned users through multi-view contrastive learning. (5) Top-k highly similar users are selected as aligned users, and then the missing network topology is completed by aligned users.
**Algorithm 1:** Social network user alignment.
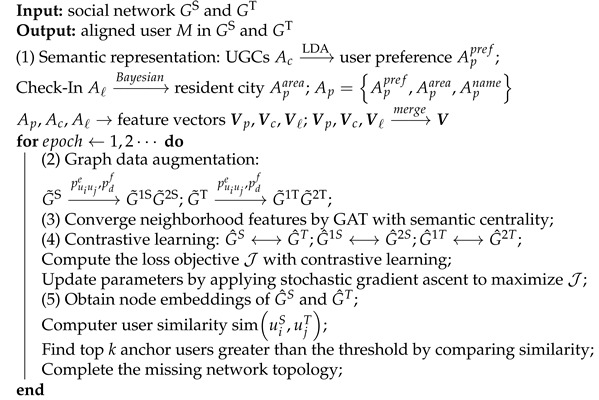


### 4.2. Multi-Level Semantic Representation

**Figure 3:**
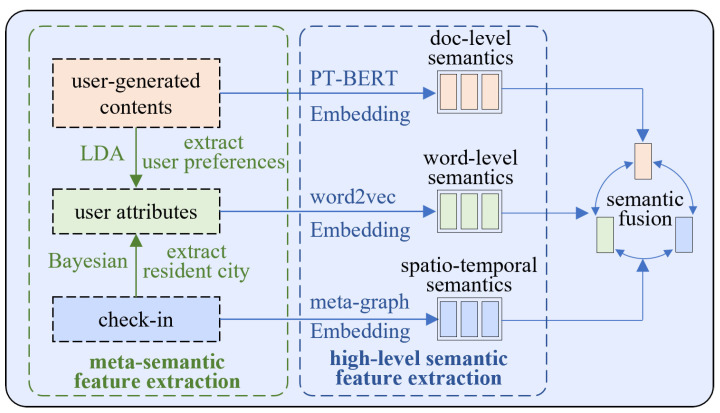
Multi-level semantic feature representation.

There are two problems with adequately representing the semantic features of users in user alignment studies. (1) Absent or fake user attributes. When users register accounts on multiple social networks for privacy protection, user attributes may be empty or forged except for the username. (2) Inadequate semantic feature mining. The embedding of user features into the low-dimensional vector space may result in some semantic features’ absence. For example, to make a computer understand human language, representing the meaning of a whole sentence with a vector will necessarily lose some semantics of the sentence. To address these two issues, we propose a multi-level semantic feature representation, outlined as shown in Figure 3.

Given two social network views to be aligned, two kind of meta-semantic features, user preferences and resident cities, are extracted from UGCs and check-ins, respectively. High-level user semantic features are extracted from three dimensions, user attributes, UGCs, and check-ins, and then embedded and fused to obtain the user’s feature embedding vector V. Feature extraction from multiple levels and dimensions can effectively enhance the semantic features of users and improve user-alignment effects.

#### 4.2.1. Meta-Semantic Feature Extraction

User attributes are highly discriminative but contain few semantic features. It is not possible to confidently conclude that two users on different social networks are the same person by looking only at the age and gender. Therefore, in this paper, users’ preferences and cities of residence extracted from UGCs and check-ins are used to supplement users’ attributes for the subsequent user alignment task. Here, user preference refers to the user’s fondness for something, and the city of residence refers to the location from which the user most frequently posts on social networks. These two meta-semantic features are extracted from UGCs and user check-ins, not filled in by users themselves; thus, they can represent user features more reliably. It can be used to compute user similarity more accurately and improve the effect of user alignment.

Extraction of User Preferences: UGCs refer to posts made by users that contain more user behavior characteristics. With the latent Dirichlet allocation (LDA) topic model, the topics of posts can be extracted from UGCs. LDA is a probabilistic topic model that analyzes the words in a document to obtain the topic of each document and its percentage. Most existing studies use a single LDA topic model for a single social network without considering the variability of users’ posts across different social networks. This approach accordingly limits the representational power when analyzing multiple social network topics. Therefore, we extract cross-view topics from the social network views to be aligned based on C-LDA [60]. The user-view and view-word distributions are employed to represent the user’s social network view preferences and the differences in language styles across different views. Each view sets a polynomial distribution of background subject words to reduce the interference generated by meaningless noise words in the document. To improve the similarity of subject terms and the association between users across social network views, we retain subject terms with high co-occurrence frequencies in different views and add them as user preferences $A_{p}^{pref}$ to user attributes $A_{p}$.

City of residence extraction: User check-ins can be used to reliably determine the times and places at which users make posts. However, the precise positioning of user check-ins in different social networks is often inconsistent, which may interfere with user alignment. If the user’s city of residence is analyzed based on the time and location of the check-in, this can fuzzify the precise location features and improve the robustness of the algorithm. Therefore, based on the Bayesian recommendation algorithm [61], we extract users’ cities of residence from multiple views based on their preferences $A_{p}^{pref}$, check-ins $A_{\ell }$, and social connections *E*. The preference-based city of residence probability is obtained based on the location of users with the same preference; the influence-based city of residence probability is obtained based on the influence of friends in the social network; the distance-based city of residence probability is obtained based on the distance between users’ check-in locations; the linear sum of these three probabilities forms the final city of residence probability. The city with the highest probability is the determined to be the city of residence of the user $A_{p}^{area}$ and is added to the user attribute $A_{p}$.

#### 4.2.2. Word-Level Semantic Representation

After meta-semantic feature extraction, user attribute $A_{p}$ includes username $A_{p}^{name}$, city of residence $A_{p}^{area}$, and interest preference $A_{p}^{pref}$. Since these words are not related to each other, global semantic features do not need to be considered. Therefore, based on word2vec [51] we vectorize the user attributes to extract word-level semantics. The user attributes are divided into words, after which, stop words (such as “a” and “the”) are dropped. Each word is represented by a Huffman encoding, making the encoding of the more frequent words shorter, which can improve the training efficiency of our algorithm. There are more repetitive words describing city and preference in user attributes, and the dataset is small, which is suitable for training word vectors with CBOW—a language model of word2vec. After the CBOW model training, we get the feature vectors $V_{p}^{name}$, $V_{p}^{area}$, and $V_{p}^{pref}$, corresponding to the username, city of residence, and interest preferences. After merging these features together, the feature vector corresponding to the user attributes is as follows:

$$V_{p}=\left[v_{1p},v_{2p},\cdots ,v_{ip},\cdots ,v_{np}\right]\in R^{D\;\times \;n},$$

where $v_{ip}$ represents the word-level semantic embedding vector of user *i*. R denotes the vector space, and *d* denotes the feature dimension of the embedding vector.

#### 4.2.3. Document-Level Semantic Representation

Compared with user attributes, UGCs contain more semantic features, such as sentiment and writing patterns. These semantic features facilitate user alignment; however, the included local semantic noise may also interfere with the alignment effect. The semantic features of UGCs cannot be fully mined using word2vec. Notably, the embedding vector trained based on the BERT [53] method contains more semantic features, which can reduce the noise information in UGCs. Therefore, based on PT-BERT [62], we extract document-level semantics from UGCs. The original sentence embedding is obtained by BERT, after which a pseudo-sentence embedding of corresponding length is generated. The original embedding and the pseudo-embedding are used to the final embedding vector based on the attention mechanism. Unbiased encoders are trained using contrast learning in true and false embedding vectors, which can enhance the semantic features of sentences. After training, the user-generated contents $A_{c}$ are converted into the corresponding feature vector:

$$V_{c}=\left[v_{1c},v_{2c},\cdots ,v_{ic},\cdots ,v_{nc}\right]\in R^{D\;\times \;n},$$

where $v_{ic}$ represents the document-level semantic embedding vector of user *i*.

#### 4.2.4. Spatiotemporal Semantic Representation

It is not easy to deeply mine the association between two users based solely on the user location at the time of posting. Therefore, we combine time and space by using ACTOR [63] to deeply mine the user’s spatio-temporal semantics and thereby improve the user-alignment effect. The times and locations of check-ins and users are used as nodes to construct a heterogeneous network. According to different types of node linkage patterns, such as T1−U1−U2−T2, temporal and spatial features are embedded into the same vector space. Deeper semantic features can be captured by maintaining the higher-order proximity of different levels. After training, the user check-in $A_{\ell }$ is transformed into a spatio-temporal semantic embedding vector:

$$V_{\ell }=\left[v_{1\ell },v_{2\ell },\cdots ,v_{i\ell },\cdots ,v_{n\ell }\right]\in R^{D\;\times \;n},$$

where $v_{I\;ell}$ represents the spatio-temporal semantic embedding vector of user *i*.

Through meta-semantic feature extraction, we obtain the user preferences $A_{p}^{pref}$ and cities of residence $A_{p}^{area}$. Adding them to the user attribute $A_{p}$ can reduce the negative impact on the user-alignment effect of missing or false of user attributes. Through multi-dimensional user feature semantic analysis, we extract the corresponding word-level feature embedding vector $V_{p}$, document-level feature embedding vector $V_{c}$, and spatio-temporal feature embedding vector $V_{\ell }$ from the user attributes $A_{p}$, user-generated contents $A_{c}$, and user check-ins $A_{\ell }$. These feature embedding vectors are fused and averaged to obtain the embedding vector V, representing user features. Meanwhile, the original views of the source and target social networks are converted to embedded views. The process is as follows:

$$G^{S}=\left(U^{S},E^{S},A^{S}\right)\to {\tilde{G}}^{S}=\left(U^{S},E^{S},V^{S}\right).$$

$$G^{T}=\left(U^{T},E^{T},A^{T}\right)\to {\tilde{G}}^{T}=\left(U^{T},E^{T},V^{T}\right).$$

### 4.3. Graph-Data Augmentation with Semantic Noise Adaption

Data augmentation is a kind of data expansion and enhancement method. In the field of image processing, data augmentation refers to increasing the sample size by transforming the image. In graph networks, graph-data augmentation is achieved by adding perturbations to edges and features [64]. Across different social networks, the semantic features and topologies of users exhibit some variability. The semantic noise included in the social network view reduces the accuracy of user alignment. To address this problem, we improve the generalization capability of the algorithm by employing graph-data augmentation for the semantic features and topologies of the users in the embedded view. The existing graph-data augmentation methods are not well adapted to the dynamic data diffusion characteristics in social networks, meaning that the user alignment is insufficiently effective. Therefore, we propose a graph-data augmentation method with a semantic centrality attention mechanism to ensure a reasonable distribution of augmentation weights. This enables the augmented view to improve the algorithm’s self-adaptation to noise while ensuring that important topologies and features remain unchanged. Below, we describe the aspects of semantic centrality, topology-level semantic augmentation, and feature-level semantic augmentation.

#### 4.3.1. Semantic Centrality

Users who communicate more frequently on social networks tend to be more semantically similar. Based on the similar semantics of friends, the semantic features of users themselves can be made more complete, which can improve user alignment. Users’ influence and preferences each have a significant impact on their communication. Due to the power-law distribution characteristic of social networks, most users usually have a small number of friends. Users who are followed by more people tend to have more influence. Moreover, users with similar preferences communicate with each other more frequently. Therefore, we compute semantic centrality attention weights based on influence and preference. The critical user features and network topology in a given view can be retained by increasing the probability of masking the features of users with low influence and the probability of removing the topology of users with different preferences.

**Figure 4:**
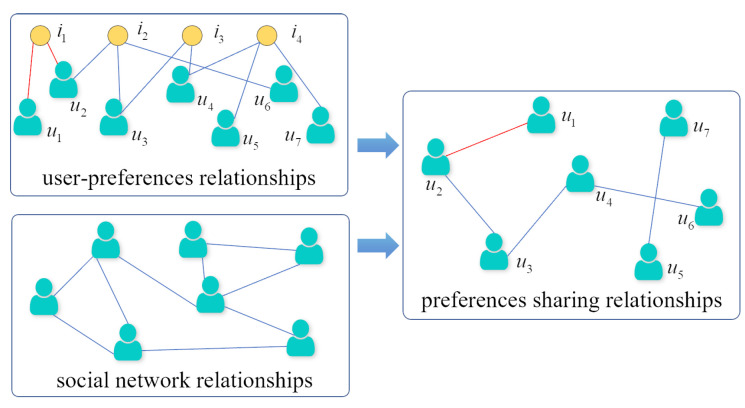
Construction process of preferences a sharing relationship.

In undirected graphs, degree indicates the number of friends of a user. We use degree centrality to indicate the importance of a user in a social network. The computation formula is as follows:

(1) $$Degree\;\;Centrality=\frac{k_{i}}{N-1},$$

where $k_{i}$ denotes the degree of user *i* and *N* denotes the total number of users in this network. The degree centrality of users in the social network relationship graph measures user influence, and the degree centrality of users in the preference sharing relationship graph measures the degree of user preference. Preference sharing relationships are constructed from user-preference relationships and social network relationships [65]. As shown in Figure 4, the network topology *E* of the embedded view represents the social network relationships. The user-preference relationships $R_{pref}$ are constructed according to the user and the corresponding user preferences $A_{p}^{pref}$. According to the user preferences, users with common preferences are constructed as preference sharing relationships. The formula can be expressed as follows:

(2) $$R_{shar}=\left(R_{pref}R_{pref}^{T}\right)\circ E$$

where *E* denotes social relationships, and the Hadamard product ∘E is used to ensure that the constructed preference sharing relationships belongs to a subset of *E*. The matrix $R_{pref}R_{pref}^{T}$ multiplied together can link users with the same preferences.

The semantic centrality $\xi \left(u_{i}\right)$ of user $u_{i}$ can be represented as

(3) $$\xi \left(u_{i}\right)=deg_{user}\left(u_{i}\right)+deg_{shar}\left(u_{i}\right)$$

where $deg_{user}\left(\cdot \right)$ denotes the degree centrality of user $u_{i}$ in the social network relationship graph, and $deg_{shar}\left(\cdot \right)$ denotes the degree centrality of user $u_{i}$ in the preference sharing relationship graph.

#### 4.3.2. Topology-Level Semantic Augmentation

Users’ friendships are inconsistent across social networks, and this topological noise can lead to semantic gaps for the same user from different views. We accordingly perform topology-level semantic augmentation based on the semantic centrality of the user, which constructs a new edge set $\tilde{E}$ from the network topology *E* of the embedded view with sampling probability $p_{u_{i}u_{j}}^{e}$. This reduces the influence of network topology noise on user alignment. The sampling probability $p_{u_{i}u_{j}}^{e}$ refers to the probability of preserving the topology $\left(u_{i},u_{j}\right)$, which reflects the importance of the edge that connects user $u_{i}$ and user $u_{j}$.

We indicate the degree of topological importance based on the average of the semantic centrality of users $u_{i}$ and $u_{j}$. The weight of the topology is the average of the semantic centrality of the connected users, namely, $w_{u_{i}u_{j}}^{e}=\left(\xi \left(u_{i}\right)+\xi \left(u_{j}\right)\right)/2$. To reduce the effect of the power-law distribution property of the social network on the drop probability, we take the logarithms of topological weights, namely, $\lambda_{u_{i}u_{j}}^{e}=\log w_{u_{i}u_{j}}^{e}$. The probabilities are normalized by the following equation:

(4) $$p_{u_{i}u_{j}}^{e}=\min\left(\frac{\lambda_{u_{i}u_{j}}^{e}-\lambda_{\min}^{e}}{\lambda_{\max}^{e}-\lambda_{\min}^{e}},p_{\tau }^{e}\right)$$

where $\lambda_{\max}^{e}$ and $\lambda_{\min}^{e}$ denote the maximum and minimum values of the topological weights $w_{u_{i}u_{j}}^{e}$, respectively. $p_{\tau }^{e}$ is the truncation probability, indicating that the topology is not allowed to fall below the probability $p_{\tau }^{e}$; this prevents damaging the topology of the network with lower sampling probability.

#### 4.3.3. Feature-Level Semantic Augmentation

The contents of users’ posts for the same event are inconsistent across social network views. This feature noise can cause semantic gaps for the same user in different views. Based on the user’s semantic centrality, feature-level semantic augmentation can reduce the negative impact of feature noise on user alignment. We zero out certain dimensions of users’ features that are unimportant, which improves the algorithm’s adaptability to feature noise. To ensure randomness, we obtain $\tilde{m}\in {\left\{0,1,\right\}}^{F}$ by randomly sampling from a vector of Bernoulli distribution with probability $1-p_{d}^{f}$, and then generate the feature vectors $\tilde{V}$. The computing process is as follows:

(5) $$\tilde{V}={\left[v_{1}\circ \tilde{m},v_{2}\circ \tilde{m},\cdots ,v_{n}\circ \tilde{m}\right]}^{T}$$

where $v_{n}$ denotes the corresponding feature vector of user *n*. The symbol ∘ is the Hadamard product, which denotes that the user features and the random vector $\tilde{m}$ are multiplied by elements.

To ensure that the generated feature vector $\tilde{V}$ retains the important user semantic features, we compute the weight of a certain dimension feature based on the semantic centrality. If the *d*-th dimension feature frequently appears in user features with high semantic centrality, then the weight of that dimension is higher. The computational formula is as follows:

(6) $$w_{d}^{f}=\sum_{u\in U}\left|v_{ud}\right|\cdot \xi \left(u\right),$$

where $v_{\mathrm{ud}}$ denotes the feature value of the *d*-th dimension of user *u* in the embedded view. The larger the absolute value, the more important the feature of the dimension.

To reduce the order of magnitude effect of high weight dimensions on low weight dimensions, we take the logarithms of the weights of the features, namely, $\lambda_{d}^{f}=\log w_{d}^{f}$. The probabilities are normalized by the following equation:

(7) $$p_{d}^{f}=1-\min\left(\frac{\lambda_{d}^{f}-\lambda_{\min}^{f}}{\lambda_{\max}^{f}-\lambda_{\min}^{f}},p_{\tau }^{f}\right),$$

where $\lambda_{\max}^{f}$ and $\lambda_{\min}^{f}$ denote the maximum and minimum values, respectively, of the *d*-dimensional feature weights. $p_{\tau }^{f}$ is the feature truncation probability, indicating that masking features are not allowed above the probability $p_{\tau }^{f}$, which prevents corrupting the user features of the embedded view.

The probabilities of topology-level semantic augmentation and feature-level semantic augmentation are stochastic. The embedded view $\tilde{G}$ generates two augmented views ${\tilde{G}}^{1}$, and ${\tilde{G}}^{2}$, after two rounds of random graph-data augmentation. The topology and features of both views are distinct, which can improve the algorithm’s ability to adapt to noise. The augmentation process of the embedded view is as follows:

$${\tilde{G}}^{S}=\left(U^{S},E^{S},V^{S}\right)\to \left\{\begin{array}{c} {\tilde{G}}^{1S}=\left(U^{S},E^{1S},{\tilde{V}}^{1S}\right)\\ {\tilde{G}}^{2S}=\left(U^{S},E^{2S},{\tilde{V}}^{2S}\right) \end{array}\right.\;;$$

$${\tilde{G}}^{T}=\left(U^{T},E^{T},V^{T}\right)\to \left\{\begin{array}{c} {\tilde{G}}^{1T}=\left(U^{T},E^{1T},{\tilde{V}}^{1T}\right)\\ {\tilde{G}}^{2T}=\left(U^{T},E^{2T},{\tilde{V}}^{2T}\right) \end{array}\right.\;;$$

### 4.4. Multi-Head Attention Semantic Fusion

The effect of user alignment depends on the similarity of the aligned users. If there is lower semantic similarity among the aligned users, the accuracy of the algorithm will be reduced. Due to the variability of user features in social networks, the extracted semantic features cannot accurately represent users. Moreover, users and friends often share similar semantic features with each other. Therefore, we implement feature-topology adaptation fusion using a multi-head graph attention network. GAT [66] can adaptively fuse social network topology and neighbor features with different weights, and also further mine users’ semantic features deeply based on a multi-head mechanism. We combine semantic centrality and GAT to increase the weight of fusing similar neighbor features. This can improve the accuracy of user alignment. This approach merges the semantic features of neighbors to enhance the features of the nodes themselves and improve the accuracy of user alignment. As some users may have excessive numbers of friends in the augmented view, fusing more neighbor features using an ordinary GNN trends to give rise to an overfitting phenomenon. Therefore, we use GAT to fuse the semantic features of our neighbors.

The semantic features of users are already available in the embedding view and the corresponding augmented view. We use $v_{i}$ and $v_{j}$ to denote the embedding vectors of users $u_{i}$ and $u_{j}$, respectively. The attention factor for these two users is computed as follows.

(8) $$e_{ij}=LeakyReLU\left(\alpha \left(Wv_{i},Wv_{j}\right)\xi \left(u_{j}\right)\right).$$

This coefficient reflects the importance of user $u_{j}$ to user $u_{i}$. In the equation, we use a linear transformation with parameters $W\in R^{D^{\prime }\times D}$, along with a self-attentive mechanism α to adaptively adjust the weights. To preserve important features, the user’s semantic centrality $\xi \left(u_{j}\right)$ is used to measure the importance of its neighbors. Finally, a nonlinear layer LeakyReLU is added to serve as the activation function. To facilitate the comparison of attention weights across users, we normalize the attention of our neighbor $u_{j}$ using the softmax function:

(9) $$\alpha_{ij}=softmax_{j}\left(e_{ij}\right)=\frac{exp(e_{ij})}{\sum_{u_{k}\in \tilde{N}\left(u_{i}\right)}exp\left(e_{ik}\right)},$$

where $\tilde{N}\left(u_{i}\right)$ is the first-order neighbor of user $u_{i}$.

To improve the semantic fusion capability of the GAT, we use *K* independent attention heads for computation and concatenation. The computation process is as follows.

(10) $$v_{i}^{{}^{\prime }}=∥{}_{k\;=\;1}^{K}\sigma \left(\sum_{u_{j}\in \tilde{N}\left(u_{i}\right)}\alpha_{ij}^{\left(k\right)}W^{\left(k\right)}v_{j}\right),$$

where ‖ indicates that the splicing operation is utilized in the features, and *K* indicates the number of heads in the multi-head attention.

The averaging operation is used at the final level. The computation process is as follows:

(11) $$v_{i}^{{}^{\prime }}=\sigma \left(\frac{1}{K}\sum_{k\;=\;1}^{K}\sum_{u_{j}\in \tilde{N}\left(u_{i}\right)}\alpha_{ij}^{\left(k\right)}W^{\left(k\right)}v_{j}\right).$$

The embedded view and the corresponding augmented view are semantically fused and constructed as a contrastive view, which facilitates the usage of contrastive learning among the views in the next section. The specific view transformation process is as follows:

$$\left\{\begin{array}{c} {\tilde{G}}^{S}=\left(U^{S},E^{S},V^{S}\right)\to {\hat{G}}^{S}=\left(U^{S},E^{S},{\hat{V}}^{S}\right)\\ {\tilde{G}}^{T}=\left(U^{T},E^{T},V^{T}\right)\to {\hat{G}}^{T}=\left(U^{T},E^{T},{\hat{V}}^{T}\right) \end{array}\right.\;;$$

$$\left\{\begin{array}{c} {\tilde{G}}^{1S}=\left(U^{S},E^{1S},{\tilde{V}}^{1S}\right)\to {\hat{G}}^{1S}=\left(U^{S},E^{1S},{\hat{V}}^{1S}\right)\\ {\tilde{G}}^{2S}=\left(U^{S},E^{2S},{\tilde{V}}^{2S}\right)\to {\hat{G}}^{2S}=\left(U^{S},E^{2S},{\hat{V}}^{2S}\right) \end{array}\right.\;;$$

$$\left\{\begin{array}{c} {\tilde{G}}^{1T}=\left(U^{T},E^{1T},{\tilde{V}}^{1T}\right)\to {\hat{G}}^{1T}=\left(U^{T},E^{1T},{\hat{V}}^{1T}\right)\\ {\tilde{G}}^{2T}=\left(U^{T},E^{2T},{\tilde{V}}^{2T}\right)\to {\hat{G}}^{2T}=\left(U^{T},E^{2T},{\hat{V}}^{2T}\right) \end{array}\right.\;;$$

### 4.5. Multi-View Contrastive Learning

Computing the similarity of users requires the semantic features of users to be embedded in the Euclidean space. The effect of generated embedding vectors on user alignment depends not only on the differential semantics of the same social network, but also on the similar semantics of the aligned users in the social network to be aligned. Therefore, we perform comparison learning across in multiple comparison views. The similar features of aligned users and the different features of non-aligned users are compared in order to optimize the embedding effect, achieve user semantic feature enhancement, and improve the alignment accuracy.

We apply contrastive learning to three pairs of views: (1) source contrastive views ${\hat{G}}^{1S}$ and ${\hat{G}}^{2S}$ generated by the source social network; (2) target contrastive views ${\hat{G}}^{1T}$ and ${\hat{G}}^{2T}$ generated by the target network; (3) source-target contrastive views (alignment views) ${\hat{G}}^{S}$ and ${\hat{G}}^{T}$, constructed by the source and target social networks. In contrastive learning, it is necessary to construct positive and negative samples, which include positive samples, inter-view negative samples, and intra-view negative samples. The following description is based on the source comparison views ${\hat{G}}^{1S}$ and ${\hat{G}}^{2S}$. As these two contrastive views are constructed based on the source social network and the set of users is unchanged, we construct $u_{i}^{1S}$ and $u_{i}^{2S}$, which belonging to the same real user as positive sample pairs. The user $u_{i}^{1S}$ and the other users of the contrastive view ${\hat{G}}^{2S}$ are constructed as inter-view negative sample pairs; and the user $u_{i}^{1S}$ and the other users of the contrastive view ${\hat{G}}^{1S}$ are constructed as intra-view negative sample pairs. The positive and negative samples of the target contrastive view ${\hat{G}}^{1T}$ and ${\hat{G}}^{2T}$ are constructed in the same way as the source contrastive view. To make the embedding vectors of aligned users more similar, we use the aligned users in the aligned views ${\hat{G}}^{S}$ and ${\hat{G}}^{T}$ as positive samples.

The contrastive views of the same social network perform contrastive learning to enhance the differential features of different users. The alignment views perform contrastive learning to enhance the similar features of known aligned users. This method effectively reduces the semantic gap and improves the alignment accuracy. By constructing the loss function based on InfoNCE Loss [64], we aim to improve the mutual information of positive samples as the goal of contrastive learning, which makes the positive sample pairs more similar. The loss function L of a positive sample pair $\left(u_{i}^{\varphi },u_{i}^{\gamma }\right)$ can be defined as follows:

(12) $$\begin{array}{c} L\left(u_{i}^{\varphi },u_{i}^{\gamma }\right)=\log\frac{e^{\theta (u_{i}^{\varphi },u_{i}^{\gamma })/\tau }}{\underset{\mathrm{positive}\;\mathrm{pair}}{\underbrace{e^{\theta (u_{i}^{\varphi },u_{i}^{\gamma })/\tau }}}+\underset{\mathrm{inter}-\mathrm{view}}{\underbrace{\sum_{k\neq i}e^{\theta (u_{i}^{\varphi },u_{k}^{\gamma })/\tau }}}+\underset{\mathrm{intra}-\mathrm{view}}{\underbrace{\sum_{k\neq i}e^{\theta (u_{i}^{\varphi },u_{k}^{\varphi })/\tau }}}};\\ s.t.\left(\varphi ,\gamma \right)=\left\{\left({\hat{G}}^{S},{\hat{G}}^{T}\right),\left({\hat{G}}^{1S},{\hat{G}}^{2S}\right),\left({\hat{G}}^{1T},{\hat{G}}^{2T}\right)\right\}. \end{array}$$

We set a temperature coefficient τ to adjust the penalty strength of the inter-view and intra-view negative sample pairs, which prevents the user alignment model from falling into a local optimum solution in training. $\theta \left(u^{1},u^{2}\right)=s\left(g\left(u^{1}\right),g\left(u^{2}\right)\right)$ is used to compute the user similarity.

The loss function $L\left(u_{i}^{\varphi },u_{i}^{\gamma }\right)$ is computed for the loss of users in the contrastive views ${\hat{G}}^{S},{\hat{G}}^{1S},{\hat{G}}^{1T}$. Since the positive samples of the contrastive views ${\hat{G}}^{S}$ and ${\hat{G}}^{T}$ are aligned users and the positive samples of the other two pairs of contrastive views are the same users, these three pairs of contrastive views can be viewed as mirror-symmetric. Therefore, the loss of the contrastive views ${\hat{G}}^{T},{\hat{G}}^{2S},{\hat{G}}^{2T}$ can be defined as $L\left(u_{i}^{\gamma },u_{i}^{\varphi }\right)$. Our overall objective is to maximize the mean of all positive sample pairs. Accordingly, the overall loss function J is computed as follows.

(13) $$J=\frac{1}{2N}\sum_{i=1}^{N}\left[L\left(u_{i}^{1},u_{i}^{2}\right)+L\left(u_{i}^{2},u_{i}^{1}\right)\right],$$

In this section, we continually reduce the value of this loss function to optimize the embedding vectors of the contrastive views ${\hat{G}}^{S}$ and ${\hat{G}}^{T}$. User-alignment accuracy can be improved by enhancing the similar semantic features of aligned users and the difference features of non-aligned users.

### 4.6. User Alignment

In this section, we compute the user similarity based on the embedding vectors of views ${\hat{G}}^{S}$ and ${\hat{G}}^{T}$. If the similarity reaches the alignment threshold, the two users of different social networks are determined to be the same real-world user. The cosine distance is used to measure the similarity of users $u_{i}^{S}$ and $u_{j}^{T}$. The calculation formula is as follows:

(14) $$\mathrm{sim}\left(u_{i}^{S},u_{j}^{T}\right)=\frac{{\hat{V}}_{i}^{S}\cdot {\hat{V}}_{j}^{T}}{∥{\hat{V}}_{i}^{S}∥∥{\hat{V}}_{j}^{T}∥},$$

where ${\hat{V}}_{i}^{S}$ and ${\hat{V}}_{i}^{T}$ denote the feature vectors of users $u_{i}$ and $u_{j}$ in the aligned views ${\hat{G}}^{S}$ and ${\hat{G}}^{T}$, respectively.

Based on the user similarity equation, we can compute the similarity of all users in the two social networks and represent them by the matrix $V^{sim}$. If $V_{ij}^{sim}$ is greater than the alignment threshold, users $u_{i}$ and $u_{j}$ are considered to be aligned users.

To make better use of the inter-layer link relationships, we add the top *k* similar aligned users that reach the similarity threshold to the known aligned user pairs *M*. Suppose there are two pairs of aligned users who are friends in the source network, but no link between them has been established in the target network; we can then complement the missing topology of the target network based on the aligned users. This can enhance user semantic features and improve user-alignment accuracy.

## 5. Experiments

Experiments were conducted on real-world social networks to evaluate the effectiveness of the proposed SENUA model when dealing with the user alignment problem. Moreover, an ablation study and comparisons of similarity before and after the experiment are conducted and discussed.

### 5.1. Dataset and Experimental Setup

#### 5.1.1. Dataset

To prove the effectiveness of the algorithm, the Douban–Weibo datasets [43] and DBLP17-DBLP19 datasets [44] are used to validate the experiment. Douban–Weibo dataset contains social network topology, user attributes, and user-generated contents. DBLP is a computer science bibliography that includes author’s name, school, city, and papers. The statistics are presented in Table 2.

**Figure 5:**
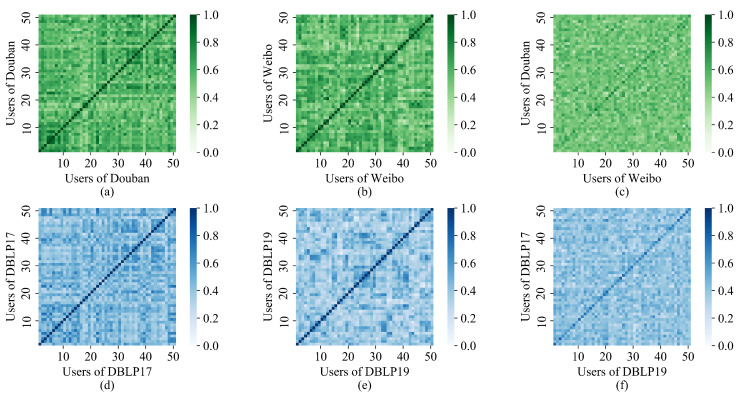
Visualization of user similarity before training: (**a**) Douban; (**b**) Weibo; (**c**) Douban-Weibo; (**d**) DBLP17; (**e**) DBLP19; (**f**) DBLP17-DBLP19.

Similar users in the same social network and similar users across social networks can affect alignment. To visualize the interference, we took 50 pairs of aligned users from both datasets and represent the user similarity with a heat map, as shown in Figure 5. Green represents the Douban–Weibo datasets, and blue represents the DBLP17-DBLP19 datasets. The labels of the 6 subgraphs indicate the social networks in which users are registered. The scale of the coordinate axis represents the user ID. Figure 5a,b,d,e represent the comparison of users in the same social network. The users of the horizontal and vertical axes are in accordance. Figure 5c,f show the comparison of aligned users in the social network to be aligned. The diagonal line indicates the similarity of the aligned user pairs. The deeper the color in the graph, the higher the degree of similarity. The figure shows that there are a large number of highly similar users in the same social network, which can interfere with user alignment. Compared with Douban–Weibo, the interference user color is lighter and the alignment user color is deeper in DBLP17-DBLP19. It is easier to achieve user alignment in DBLP17-DBLP19 datasets. We aimed to improve the color depth of the diagonal lines in Figure 5c,f. Increasing the color depth of the diagonal in Figure 5c,f is our goal. We ensured the accuracy of user alignment by reducing noise in social networks and optimizing embedding effects.

#### 5.1.2. Parameter Settings

After treating the following–followed relationship as an undirected edge, we expand the directed edges of the dataset into undirected edges. We extracted user semantic features from user attributes, UGCs, and user check-ins with an embedding dimension of 256. The projection before user alignment comprises two fully connected layers, where the hidden dimension was 512. The edge sampling probability in graph augmentation was 0.3. The feature masking probability was 0.2. The temperature parameter τ was 0.2 for contrastive learning based on InfoNCE.

#### 5.1.3. Evaluation Indicators

Hit-Precisionk was used as the performance metric for this experiment. This metric represents the average score of the top *k* positive samples in the prediction results, which can represent the prediction accuracy of our algorithm. The computation formula is as follows:

(15) $$\;\;\mathrm{Hit}-\mathrm{Precision}@k=\frac{1}{\left|C\right|}\sum_{x\in C}\frac{k-(\mathrm{hit}(x)-1)}{k},$$

where C indicates the set of candidate users, and hit(x) indicates the location of the positive sample among the top-*k* recommended candidate users.

### 5.2. Baseline Methods

To verify the performance of this algorithm, we chose the following user alignment algorithms as the baselines.

- GraphUIL [21] encodes the local and global network structures, then achieves user alignment by minimizing the difference before and after reconstruction and the match loss of anchor users.
- INFUNE [43] performs information fusion based on the network topology, attributes, and generated contents of users. Adaptive fusion of neighborhood features based on a graph neural network is performed to improve user-alignment accuracy.
- MAUIL [44] uses three layers of user attribute embedding and one layer of network topology embedding to mine user features. User alignment is performed after mapping user features from two social networks to the same space.
- SNAME [67] effectively mines user features based on three embedding methods: intentional neural network, fuzzy c-mean clustering, and graph drawing embedding.

### 5.3. Experimental Results

**Figure 6:**
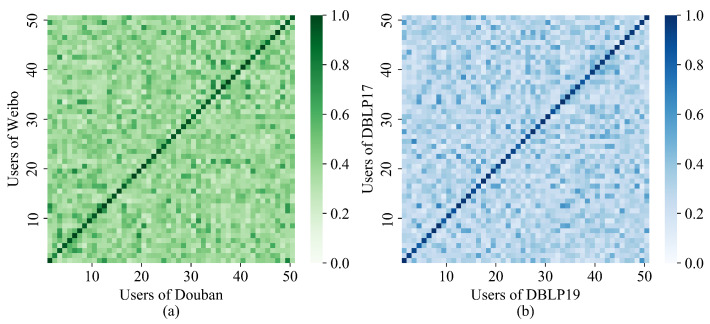
Visualization of trained user similarity: (**a**) Douban-Weibo; (**b**) DBLP17-DBLP19.

**Figure 7:**
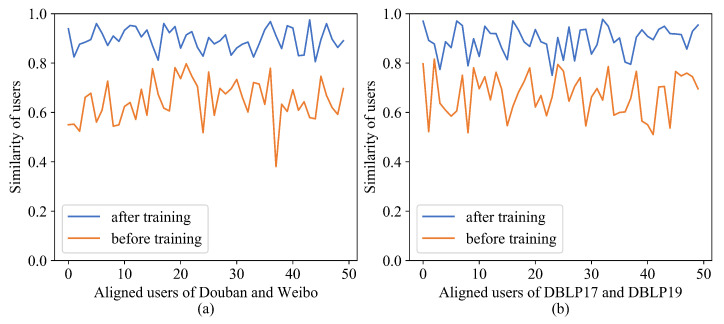
Comparison of user similarity before and after training: (**a**) Douban-Weibo; (**b**) DBLP17-DBLP19.

Figure 6 presents the heat map of user similarity of two datasets after SENUA training. The diagonal lines indicate the similarity of aligned users, and the other regions indicate the similarity of non-aligned users. Compared with the pretraining Figure 5c,f, it can be observed that the diagonal colors are significantly deeper, and the colors of the remaining positions are significantly lighter. Overall, SENUA reduces the interference of highly similar users on the user-alignment effect and accordingly improves the alignment effect. Figure 7 presents the similarity comparison of aligned users before and after training. The horizontal axis represents the users to be aligned, and the vertical axis is the user similarity. As the figure makes clear, the similarity of aligned users is significantly improved after training, and the similarity changes are more stable. The multi-head attention semantic fusion makes the embedding vector more stable, and contrastive learning in aligned views enhances the similarity of aligned users, which plays an important role in improving user alignment accuracy.

**Figure 8:**
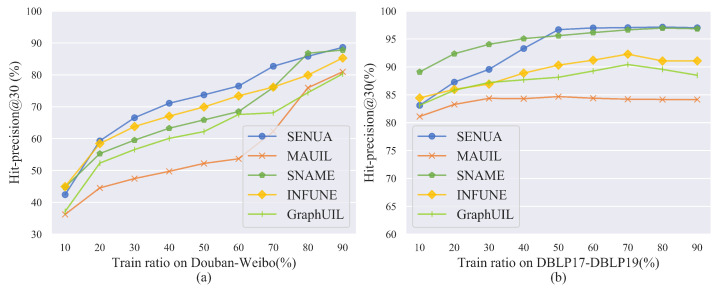
Comparisons with baselines: (**a**) Douban-Weibo; (**b**) DBLP17-DBLP19.

To demonstrate the effectiveness of our algorithm, we compare the user-alignment accuracy of each algorithm based on the Douban–Weibo and DBLP17-DBLP19 datasets, as shown in Figure 8. The horizontal axis is the ratio of the training set to the total dataset. The vertical axis is the performance metric hit-precision30, which indicates the existence probability of aligned users among the 30 similar users recommended for the user. This can represent the prediction accuracy of aligned users in different social networks. The results show that SENUA outperformed other baseline methods in user alignment, with an average improvement of 6.27%. This shows that multi-view graph contrastive learning can improve the effectiveness of social network user alignment. The overall performance in Figure 8b is significantly better than that in Figure 8a. User alignment can also achieve better results when the training ratio of DBLP is 10%. In Figure 5d,e, there are fewer highly similar users in the same social network, and the user alignment is less affected by noise interference. Compared with Figure 5c, the diagonal line of Figure 5f is darker, and other areas are lighter in color. In the DBLP17-DBLP19 dataset, the aligned users are subject to less interference, which results in better user alignment in this dataset. Our algorithm is not optimal when the training ratio is 10%. As the training ratio increases, the alignment accuracy continues to improve. Better user alignment is obtained when the training ratio is high. Graph attention networks and contrastive learning all require sufficient data to accurately discover the feature patterns of users. We reduce the local noise interference by multi-level user feature representation, and then effectively enhance the semantic features of users by semantic fusion and semantic contrasting.

**Figure 9:**
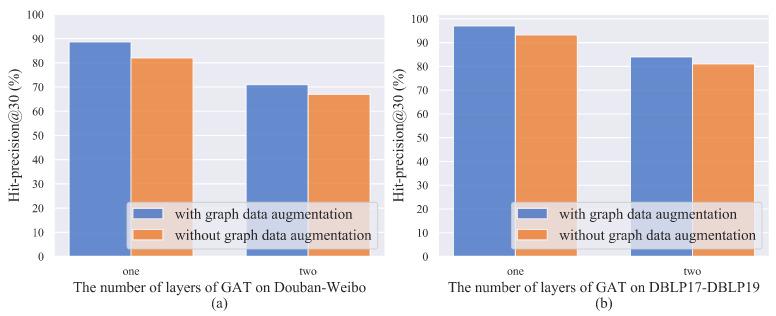
The impacts of GAT and graph-data augmentation on user alignment: (**a**) Douban-Weibo; (**b**) DBLP17-DBLP19.

We fixed the ratio of the training set to the total dataset to 0.9. Subsequently, the effects of GAT and graph-data augmentation on user alignment were measured, as shown in Figure 9. The accuracy decreases slightly at one layer of GAT and without graph-data augmentation. The graph-data augmentation improves the peak accuracy of our algorithm, although the impact on the accuracy is small. With semantic centrality attention, graph-data augmentation can reduce noise interference in social networks while preserving important features and topology. After the number of layers of GAT is adjusted from one to two, the user-alignment accuracy decreases significantly. If the number of GAT layers is too high, users will fuse more neighborhood features, which will reduce the feature variability among users and lead to difficulties in user alignment.

## 6. Conclusions

In this paper, we proposed a semantic-enhancement-based social network user alignment algorithm, SENUA, to reduce the semantic-gap problem caused by social network variability. The interference of local semantic noise on user alignment is reduced through the use of multi-level semantic representations. To reduce the feature noise and topological noise in the aligned views, we improved the algorithm’s ability to adapt to semantic noise by using graph-data augmentation. Appropriate weights are assigned to the user’s semantic features and topology with the semantic centrality of the user, which enables important semantic features to be preserved. The embedding vectors of users are optimized based on multi-head graph attention networks and multi-view contrastive learning. By increasing the embedding distance between users in the same social network views while decreasing the embedding distance of aligned users in the aligned views, we can effectively enhance the semantic features of users and improve the alignment effect. To verify the performance of our model, we compared it with several baseline methods on the Douban–Weibo and DBLP17-DBLP19. Experimental results show that the effectiveness of SENUA is 6.27% higher than that of the baseline methods on average. As these results show, SENUA enhances user alignment through semantic enhancement in many ways. However, semantic fusion and multi-view contrastive learning generate a high computing overhead. In our future work, we plan to improve the efficiency and accuracy of user alignment based on causal inference.

## Figures and Tables

**Table 1:** Definitions of symbols.

Notation	Definition
$G^{S}$,$G^{T}$	Source social network, target social network.
*U*	Set of users in the social network.
*E*	Edge set of the social network.
*A*	User features of the social network.
$A_{p},A_{c},A_{\ell }$	User attributes, UGCs, and user check-ins.
$A_{p}^{name},A_{p}^{area},A_{p}^{pref}$	User name, city of residence, and user preference.
$u_{i}$	The *i*th user.
V	Embedding vectors of user semantic features.
R	Vector space.
*D*	Feature dimension.
*N*	Total number of users in the network.
*M*	Aligned user pairs.
$R_{shar}$	Preference sharing matrix.
$\xi \left(u_{i}\right)$	Semantic centrality of user $u_{i}$.
$p_{u_{i}u_{j}}^{e}$	Topology sampling probability.
$p_{d}^{f}$	Feature masking probability.

**Table 2:** Statistics of the datasets.

Datasets	Networks	Users	Edges	Min Degree	Ave Degree	Max Degree	Anchors	Source
Social networks	Douban	9734	200,467	1	43	1723	9514	[43]
	Weibo	9514	196,978	1	34	2501		
coauthor networks	DBLP17	9086	51,700	2	5.7	144	2832	[44]
	DBLP19	9325	47,775	2	5.1	138		

## Data Availability

Not applicable.
